# Non-Buffer Epi-AlGaN/GaN on SiC for High-Performance Depletion-Mode MIS-HEMTs Fabrication

**DOI:** 10.3390/mi14081523

**Published:** 2023-07-29

**Authors:** Penghao Zhang, Luyu Wang, Kaiyue Zhu, Qiang Wang, Maolin Pan, Ziqiang Huang, Yannan Yang, Xinling Xie, Hai Huang, Xin Hu, Saisheng Xu, Min Xu, Chen Wang, Chunlei Wu, David Wei Zhang

**Affiliations:** 1 State Key Laboratory of ASIC and System, School of Microelectronics, Fudan University, Shanghai 200433, China; phzhang19@fudan.edu.cn (P.Z.); wangly20@fudan.edu.cn (L.W.); 21112020111@m.fudan.edu.cn (Q.W.); mlpan21@m.fudan.edu.cn (M.P.); zqhuang20@fudan.edu.cn (Z.H.); yangyn20@fudan.edu.cn (Y.Y.); 21212020013@m.fudan.edu.cn (X.X.); 22212020008@m.fudan.edu.cn (H.H.); 22212020078@m.fudan.edu.cn (X.H.); ssxu@fudan.edu.cn (S.X.); wuchunlei@fudan.edu.cn (C.W.); 2 Department of Materials, Imperial College London, London SW7 2AZ, UK; fz322@ic.ac.uk

**Keywords:** GaN, MIS-HEMTs, buffer layer, SiC substrate, current collapse

## Abstract

A systematic study of epi-AlGaN/GaN on a SiC substrate was conducted through a comprehensive analysis of material properties and device performance. In this novel epitaxial design, an AlGaN/GaN channel layer was grown directly on the AlN nucleation layer, without the conventional doped thick buffer layer. Compared to the conventional epi-structures on the SiC and Si substrates, the non-buffer epi-AlGaN/GaN structure had a better crystalline quality and surface morphology, with reliable control of growth stress. Hall measurements showed that the novel structure exhibited comparable transport properties to the conventional epi-structure on the SiC substrate, regardless of the buffer layer. Furthermore, almost unchanged carrier distribution from room temperature to 150 °C indicated excellent two-dimensional electron gas (2DEG) confinement due to the pulling effect of the conduction band from the nucleation layer as a back-barrier. High-performance depletion-mode MIS-HEMTs were demonstrated with on-resistance of 5.84 Ω·mm and an output current of 1002 mA/mm. The dynamic characteristics showed a much smaller decrease in the saturation current (only ~7%), with a quiescent drain bias of 40 V, which was strong evidence of less electron trapping owing to the high-quality non-buffer AlGaN/GaN epitaxial growth.

## 1. Introduction

AlGaN/GaN high electron mobility transistors (HEMTs), as a representative of GaN-based wide-band gap semiconductor devices, are becoming one of the most promising candidates for next-generation high-density power conversion systems due to high breakdown voltage, low on-resistance, a wide range of operating temperatures, and high frequency switching [1,2]. Considering the advantages of substrate size and cost, GaN on Si technology has been widely used in AlGaN/GaN HEMTs fabrication [3]. However, large mismatches of lattice dimension and thermal expansion between Si and (Al)GaN will lead to high-density dislocation, poor crystalline quality, and even wafer cracks in the (Al)GaN epilayer due to the failure of stress control. To solve these problems, some strain management procedures have been developed such as AlN/GaN superlattice [4], gradient Al component from AlN to GaN [5,6], and an AlN insert layer [7].

Due to the presence of impurities as Si and O in the metal-organic chemical vapor deposition (MOCVD) chamber, *n*-type (Al)GaN has often been obtained for the buffer layer, even in the unintentional doping (UID) epitaxial process. Therefore, in order to reduce the vertical leakage and increase the breakdown voltage of devices [8], several methods have been proposed, such as a thicker buffer layer [9], optimized concentration of C-doping or Fe-doping, and so on [10,11,12]. It should be pointed out that a large number of deep-level traps will be introduced during compensation doping in the thick buffer layer, which is one of the main causes of the current collapse phenomenon of AlGaN/GaN HEMTs, leading to increasing dynamic on-resistance and a decreasing output current. In other words, the buffer layer on the Si substrate has both positive as well as negative effects on device performance.

Improving the epi-structure and substrate design is a promising method to enhance the dynamic performance of devices. For the 4H-SiC substrate, its lattice mismatch with (Al)GaN is much lower [13,14]; hence, there is no urgent need for the thick buffer layer to reduce dislocation. Compared with *p*-type doping Si, the 4H-SiC substrate is semi-insulating, which could suppress the vertical leakage current without the help of a high-resistance buffer. In addition, the thermal conductivity of SiC (substrate thickness of 0.5 mm) is much higher than that of Si (substrate thickness of 1 mm), indicating that there is a significant advantage in its heat dissipation capability [13]. Recently, the growth technique of SiC has been rapidly developed due to the huge demand for electric vehicles. Thus, the cost of SiC substrates will come down gradually, causing GaN on SiC to become a potential solution for high-end power-switching applications, instead of GaN on Si.

Chen [15] and Hult [16] et al. proposed an epi-structure named QuanFINE based on a SiC substrate that skipped the thick buffer layer between the AlN nucleation layer and GaN channel layer, showing some superiority in their reports, respectively. However, the properties of the epitaxial wafer and the device performance based on this novel epi-structure have not been studied in detail yet. In this work, a variety of characterization methods were employed to systematically evaluate the non-buffer epi-structure without a buffer layer compared with conventional epitaxial wafers based on SiC and Si substrates. Temperature-changing tests were performed for the Schottky diodes on the three epi-structures to further investigate the surface and transport properties of two-dimensional electron gas (2DEG). Normally-on metal insulator semiconductor-HEMTs (MIS-HEMTs) were also fabricated. The DC and dynamic performances of the devices were studied and compared, and the advantages of the non-buffer structure were revealed.

## 2. Experimental Section

**Figure 1:**
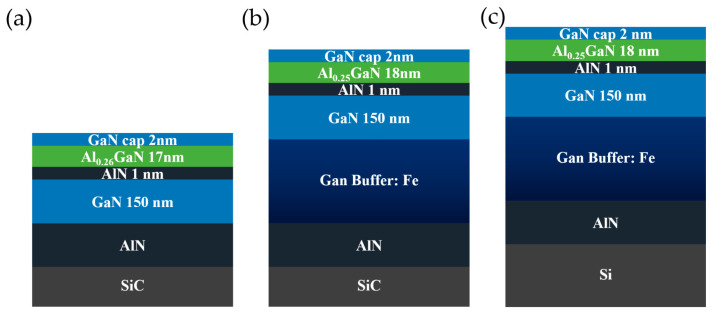
Schematic structures of (**a**) non-buffer epi-structure on SiC substrate, i.e., Epi A, (**b**) conventional epi-structure on SiC substrate, i.e., Epi B, and (**c**) conventional epi-structure on Si substrate, i.e., Epi C.

Three AlGaN/GaN hetero-structures epitaxially grown by MOCVD on 4-inch semi-insulating SiC substrates and 6-inch *p*-doping Si substrates were used in this paper. The novel epi-structure without a buffer layer was GaN (2 nm)/Al_0.26_Ga_0.74_N (17 nm)/AlN (1 nm)/UID GaN (150 nm)/AlN (200 nm)/SiC (0.5 mm). It should be pointed out that a unique epitaxy process of the AlN nucleating layer and interface treatment was developed to play an important role in the high crystal quality of the AlN growth. The other two conventional epitaxial wafers on SiC and Si substrates were grown simultaneously with the same structure: GaN (2 nm)/Al_0.25_Ga_0.75_N (18 nm)/AlN (1 nm)/UID GaN (150 nm)/Buffer (Fe-doping, 2.2 μm)/AlN (200 nm)/SiC (0.5 mm) or Si (1 mm). They are referred to as Epi A, Epi B, and Epi C in the rest of this article. The schematic structures of the above epitaxial wafers are shown in Figure 1a–c.

Four characterization methods were performed to investigate the effects of the non-buffer design on the heterojunction and surface properties. A WITec alpha300R Raman Imaging Microscope (WITec, Ulm, Germany) was used to study the growth stress with a 405 nm solid-state laser. Rigaku SmartLabII X-ray Diffraction (XRD) (Rigaku, Tokyo, Japan) was employed to characterize the crystalline quality of the epilayer through X-ray rocking curves of (002) and (102). The surface morphology was evaluated using a Park NX10 Atomic Force Microscope (AFM) (Park Systems Corp., Suwon, Republic of Korea) in no-contact mode (NCM). The carrier concentration and mobility in the heterojunction channel were measured by the Ecopia AHT55T3 (Ecopia, Anyang, Republic of Korea) Hall Effect Measurement System.

The fabrication of Schottky diodes on the three epi-structures started with mesa etch using an inductively coupled plasma (ICP) etcher with Cl-based gas. Ti/Al/Ni/Au (20/120/50/50 nm) metal stacks were deposited as the source/drain electrodes by electron beam evaporation (EBE). The ohmic contact was formed by rapid thermal annealing (RTA) at the temperature range of 790 °C to 860 °C for 30 s in N_2_ ambient, according to the different crystalline quality in the AlGaN barrier layer on different substrates. The *I*-*V* results of the transmission line model (TLM) suggested that the contact resistance (*R*_c_) of each epi-structure was 0.82 Ω·mm, 0.73 Ω·mm, and 0.77 Ω·mm, sequentially. Finally, the gate electrodes of Ni/Au (40/60 nm) were deposited by EBE.

**Figure 2:**
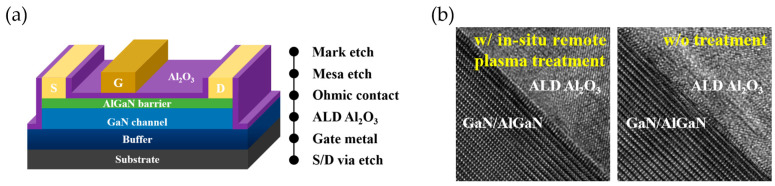
(**a**) Schematic process flow of MIS-HEMTs fabrication. (**b**) Cross-sectional TEM micrographs of the Al_2_O_3_/GaN/AlGaN interface with and without in-situ plasma treatment.

The process flow for MIS-HEMTs was similar with Schottky diodes, with the addition of a gate dielectric process between the ohmic contact formation and the gate metal deposition, as shown in Figure 2a. Before 20 nm Al_2_O_3_ deposition by thermal atomic layer deposition (ALD), in-situ remote NH_3_/N_2_ plasma treatment was carried out in a Sentech Si-500 PEALD system (Sentech, Berlin, Germany), which was applied to the gate region in sequence at an RF plasma power of 100/50 W, gas flow of 100/100 sccm, process time of 4/10 min, and substrate temperature of 300 °C. NH_3_ plasma acted as a deoxidation step to remove the native oxide, while the subsequent N_2_ plasma compensated the N vacancy beneath the surface and formed the nitridated interface. After the following ALD process, post-deposition annealing (PDA) was implemented at 450 °C in O_2_ ambient for 3 min to reduce the defects in the film. Transmission electron microscopy (TEM) was performed on the Al_2_O_3_/GaN/AlGaN interface to examine the effect of in-situ remote plasma treatment, as shown in Figure 2b. The boundary of the interface without any treatment was rough, which tended to be sharp after the low-damage in-situ NH_3_/N_2_ plasma treatment.

## 3. Results and Discussion

### 3.1. Epitaxial Wafer Quality Characterization

**Figure 3:**
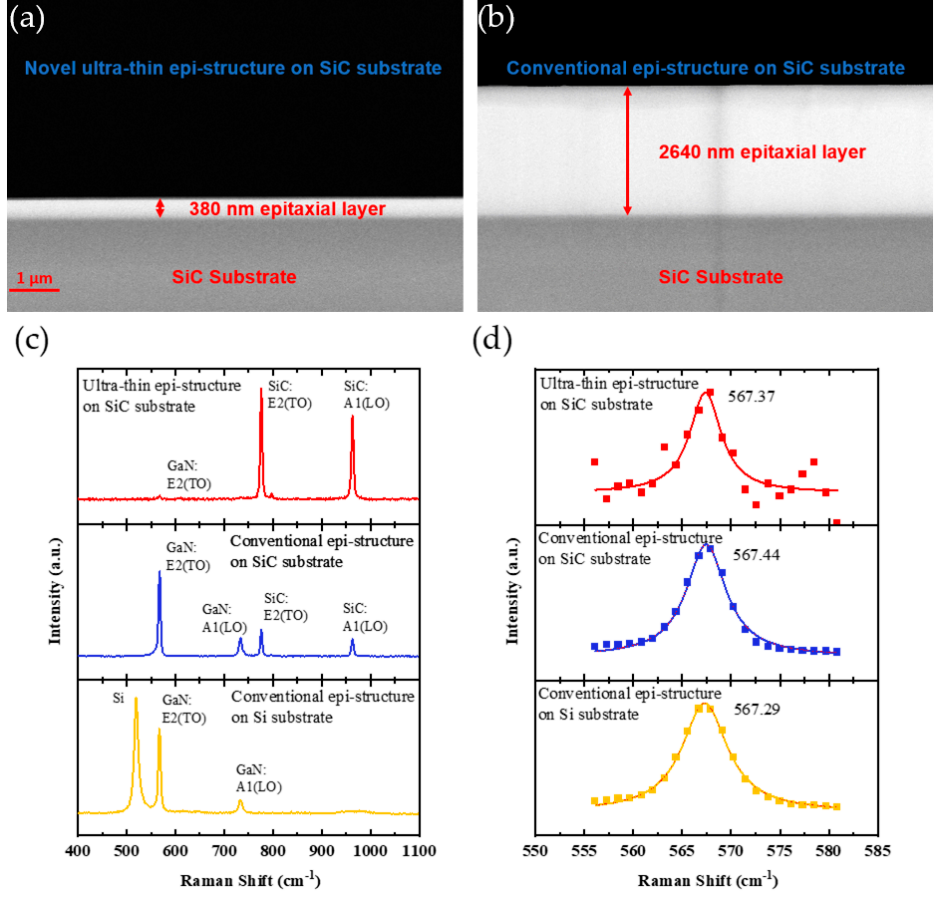
Cross-section views of (**a**) Epi A and (**b**) Epi B by SEM. (**c**) Room temperature Raman spectra of the three epi-structures on SiC and Si substrates under 405 nm laser line excitation. (**d**) Lorentz fitting curves of GaN E2(TO) for above samples.

Figure 3a,b display the cross-section views of the non-buffer epi-structure, i.e., Epi A, and conventional epi-structure on the SiC substrate, i.e., Epi B, by scanning electron microscopy (SEM). The thickness of the total epilayer on Epi A was approximately 380 nm, which was only 1/7 compared with the conventional epilayer on Epi B, indicating a clear advantage of heat dissipation of the devices. However, the surface stress on Epi A should be investigated due to the absence of a buffer layer, which is considered to play a crucial role in stress control on conventional epi-structures. The scattering peaks of GaN in E2(TO) mode could be measured by Raman spectroscopy; Raman shift is sensitive enough to characterize the growth stress for a GaN epilayer. Figure 3c shows the Raman spectrum of the above three epi-structures. The peaks of SiC, Si, and GaN exhibited different strengths, consistent with the thickness of the respective epilayers and substrates. Figure 3d shows the Lorentz fitting results of the GaN E2(TO) peak for the above samples. The Raman shifts were 567.37 cm^−1^, 567.44 cm^−1^, and 567.29 cm^−1^, respectively. Compared with the theoretical position of 567.6 cm^−1^ [17,18], slight red shifts occurred in all samples, which suggests that there was tensile stress on the surface with intensities of 0.07 GPa, 0.10 GPa, and 0.13 GPa by calculation [19]. The peak positions of GaN E2(TO) for the three epi-structures were very close to the theoretical value, indicating that the epitaxial process on the SiC substrate without a buffer layer could still achieve effective stress control.

**Figure 4:**
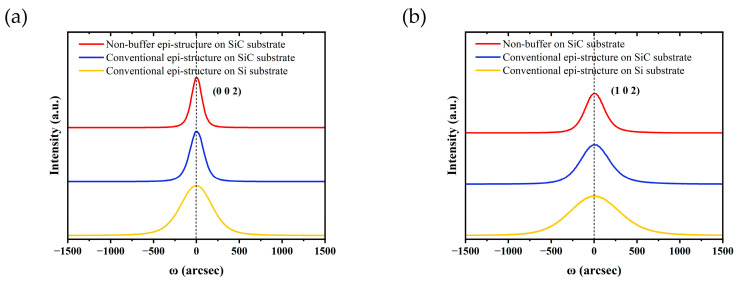
X-ray rocking curves of (**a**) (002) and (**b**) (102) for the three epi-structures by XRD.

The crystalline quality of the (Al)GaN epilayer could be characterized by the full width at half maximum (FWHM) in the XRD rocking curves of (002) and (102) planes, respectively. The FWHM of (002) and (102) represents the densities of helical and mixed dislocation [20], as shown in Figure 4a. The non-buffer epi-structure on the SiC substrate had the lowest dislocation density, with FWHMs of (002) and (102) of only 145 arcsec and 267 arcsec, confirming that the growing doping buffer layer was not essential on the SiC substrate. For the conventional epi-structure on SiC, the FWHMs were 196 arcsec and 395 arcsec. The FWHMs of the conventional epi-structure on Si were measured as 433 arcsec and 681 arcsec. This result fully indicates that the epi-structures on the 4H-SiC substrate had a lower dislocation density and higher crystalline quality than that on the Si substrate.

**Figure 5:**
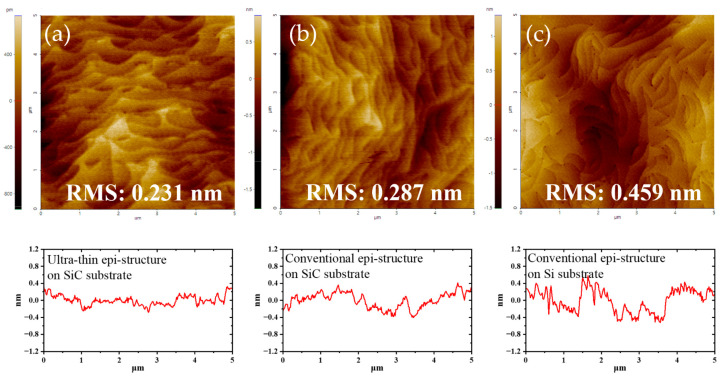
Surface morphology of (**a**) Epi A, (**b**) Epi B, and (**c**) Epi C by AFM.

Figure 5 shows the surface morphology of the above three epi-structures measured by AFM (5 × 5 μm^2^). Atomic step-flow patterns were observed in all of them. The mean square root (RMS) roughness of two epi-structures on the SiC substrate was much less than those on the Si substrate. There were plenty of pits due to dislocation at both ends of the step flow curve on the surface of the epi-structure on the Si substrate. For comparison, no clear dislocation points could be observed on the surface of the other two epi-structures on the SiC substrate. Among these epi-structures, the non-buffer one had the smallest RMS roughness of 0.231 nm, indicating the lowest density of dislocation results and best surface morphology.

Hall measurements were used to study the electrical performance of these heterojunction channels. The results are recorded in Table 1, where *R*_sheet_ is the sheet resistance of the heterojunction, *μ*_2DEG_ is 2DEG mobility, and *n*_s_ is electron density. The smallest *R*_sheet_ of 284 Ω/sq and largest *n*_s_ up to 9.8 × 10^13^ cm^−2^ were measured on the conventional epi-structure on the SiC substrate. Although owning the same epilayer, *R*_sheet_ of the conventional epi-structure on Si was nearly 30% larger. The non-buffer epi-structure on the SiC substrate had the highest *μ*_2DEG_ of 1835 cm^2^/V·s and *n*_s_ of 9.3 × 10^12^ cm^−2^, and its *R*_sheet_ was slightly higher than that for the conventional epi-structure. These results indicate that the 2DEG properties were almost independent of the buffer design but strongly related to the crystalline quality of the (Al)GaN epilayer.

### 3.2. Temperature Changing Tests for Schottky Diodes

**Figure 6:**
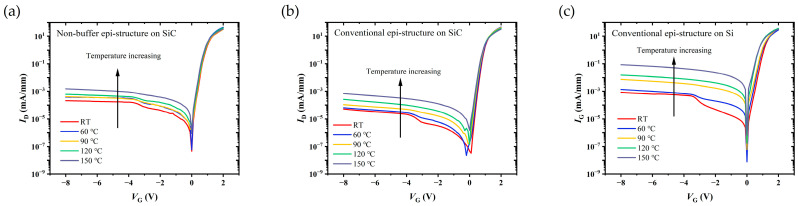
Measured *I*-*V* characteristics for Schottky diodes at different temperatures of RT ~ 150 °C on (**a**) Epi A, (**b**) Epi B, and (**c**) Epi C.

The *I*-*V* and *C*-*V* properties of the Schottky diodes on the three epi-structures were measured by an MPITS2000-SE probe platform at changing temperatures in the range of room temperature (RT) to 150 °C. The voltage scanning range on the anode was −8 V to 2 V, while the cathode was connected to the ground. As shown in Figure 6, while the temperature rose, the forward current and reverse leakage both increased on each diode. The forward current increased mainly because the high temperature improved the ability concerning 2DEG spilling over the AlGaN barrier. At 150 °C, the reverse leakage of the two epi-structures on the SiC substrate increased by approximately one order of magnitude, from ~10^−4^ mA/mm to ~10^−3^ mA/mm. The reverse current of the epi-structure on the Si substrate increased by more than 2 orders of magnitude, from ~10^−3^ mA/mm to ~10^−1^ mA/mm. The significant increase in reverse leakage at high temperatures was mainly due to the trap-assisted tunneling mechanism caused by helical dislocation [21]. The non-buffer epi-structure on the SiC substrate had the minimum reverse leakage increment at high temperatures, indicating the best quality of Schottky contact. These results prove the reliability of XRD whereby the novel epitaxial design had the smallest dislocation density.

**Figure 7:**
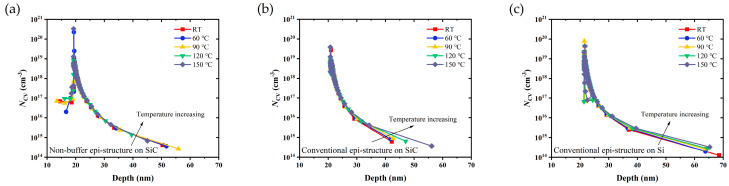
Calculated carrier distribution at different temperatures of RT ~150 °C on (**a**) Epi A, (**b**) Epi B, and (**c**) Epi C.

*C*-*V* measurements of these Schottky diodes were also carried out. Carrier distribution at different temperatures could be obtained through further calculation, as shown in Figure 7. The highest carrier concentration was found at 20~22 nm beneath the surface of the three epi-structures, which was the location of the AlGaN/GaN heterojunction channel. For the conventional epi-structures on the SiC and Si substrates, the distribution of the carrier in the direction to the channel and buffer layers was clearly widened as the temperature increased, which indicated that the high temperature led to the degradation of 2DEG confinement, and a few electrons spilled out from the channel.

**Figure 8:**
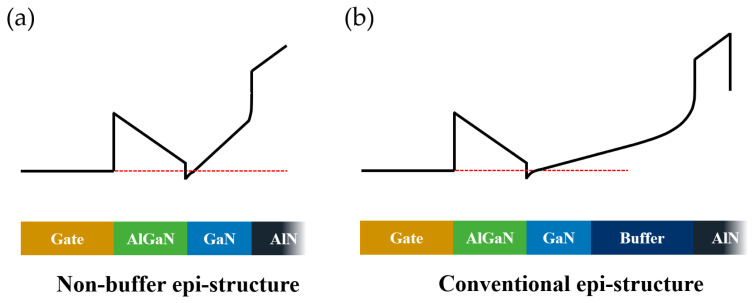
Conduction band (E_c_) (black line) of a (**a**) novel non-buffer epi-structure and (**b**) conventional epi-structure.

The following theory explains the effect of the non-buffer design on 2DEG confinement from the perspective of the energy band [15]. As shown in Figure 8, the GaN channel layer was located upon the AlN nuclear layer in the epi-structure without a buffer layer; thus, the energy band of the GaN channel layer was pulled up by AlN due to its larger band gap. AlN could serve as the back barrier of the AlGaN/GaN interface, making the back conduction band of the AlGaN/GaN heterojunction potential well very steep. However, for the conventional epi-structures, the pulling effect from the AlN nucleation layer was not clear due to the existence of a GaN buffer layer with the thickness of a micron order.

### 3.3. Device Performance of the MIS-HEMTs

**Figure 9:**
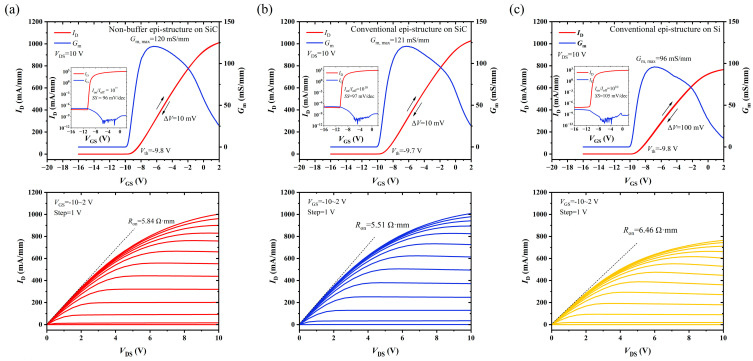
DC transfer and output characteristics for MIS-HEMTs on (**a**) Epi A, (**b**) Epi B, and (**c**) Epi C.

The fabricated MIS-HEMTs had a gate length (*L*_g_) of 4 µm, gate-source distance (*L*_gs_) of 4 µm, gate-drain distance (*L*_gd_) of 6 µm, and gate width (*W*_g_) of 50 µm. Transfer and output characteristics were measured with a parameter analyzer of Keithley 4200A (Keithley, Solon, OH, USA), as shown in Figure 9. The linear transfer characteristics were obtained for *V*_DS_ = 10 V in both forward and reverse sweep directions with *V*_GS_ steps of 0.1 V. After in-situ NH_3_/N_2_ remote plasma interface treatment, the Al_2_O_3_/GaN/AlGaN interfaces on the three epi-structures showed low interfacial state density with small hysteresis voltage Δ*V*_th_. The Δ*V*_th_ of the MIS-HEMTs on the SiC substrate were both only 10 mV, which was only 1/10 of those on the Si substrate. The transconductance (*G*_m_) of the two types of devices on the SiC substrates were ~120 mS/mm and 121 mS/mm, respectively, which was 25% higher than that on the Si substrate. MIS-HEMTs on the SiC substrate also had almost the same subthreshold swings (SSs), which were 97 mV/dec and 96 mV/dec, respectively, much less than those on the Si substrate of ~130 mV/dec.

The output characteristics were measured at *V*_DS_ up to 10 V, with *V*_GS_ in the range of −10 V to 2 V in steps of +1 V. The MIS-HEMTs on all the epi-structures exhibited kink-free characteristics. MIS-HEMTs on the SiC substrate with a thick, doped buffer showed the highest maximum current *I*_D,max_ of 1007 mA/mm and lowest on-resistance (*R*_on_) of 5.51 Ω·mm. The output characteristic of the devices with a novel non-buffer epi-structure was almost the same as that with the conventional structure, while the *I*_D,max_ and *R*_on_ of the MIS-HEMTs on the Si substrate were only 760 mA/mm and 6.46 Ω·mm, respectively.

Pulsed-*I*-*V* measurements under slow switching were performed to characterize the degree of current collapse of the fabricated MIS-HEMTs. The dynamic characteristics were investigated using the Keithley 4200A PMU module with the drain quiescent bias voltage *V*_DSQ_. The period of the square wave pulse signal was 1 ms, with a width of 10 μs, duty cycle of 1%, and time of pulse rising and falling of 500 ns. The device was synchronously switched from a quiescent bias of *V*_GSQ_ = 0 V, *V*_DSQ_ = 0/10/20/30/40 V to a measurement state of *V*_GS_ = 2 V and *V*_DS_ from 0 V to 10 V.

**Figure 10:**
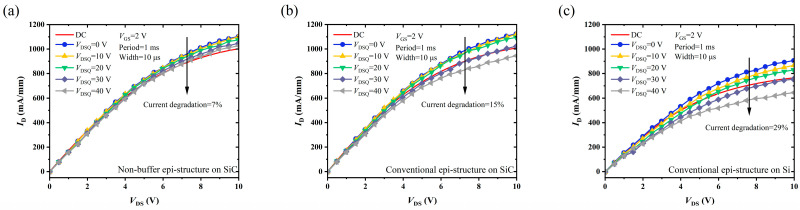
Pulsed-*I*-*V* measurements performed at different *V*_DSQ_ MIS-HEMTs on (**a**) Epi A, (**b**) Epi B, and (**c**) Epi C.

Figure 10 illustrates the current collapse phenomenon of the MIS-HEMTs on the different epi-structures under increasing *V*_DSQ_. When *V*_DSQ_ = 40 V, the current collapse of the conventional epitaxial structure on the Si substrate was 29%, which was the most serious among all the structures. For the two types of devices on the SiC substrate, no significant current collapse was observed when *V*_DSQ_ was within 20 V, indicating that the “virtual gate” effect did not occur at a weak field. When the *V*_DSQ_ was up to 40 V, the degree of current collapse on the novel non-buffer epi-structure was only 7%, while approximately 15% of current decrement occurred on the conventional structure. The non-buffer epi-structure on the SiC substrate had the best dynamic performance, which was mainly due to the following reasons: First, the novel epi-structure had a better crystalline quality and surface morphology, lower dislocation density, and resulted in fewer surface and body defects. Second, the novel structure offered better 2DEG confinement, which effectively reduced the probability of hot electron tunneling. Third, there were no deep-level traps caused by the doped thick buffer; thus, there was no contribution to current collapse.

## 4. Conclusions

In this study, a novel AlGaN/GaN epitaxial growth without a conventional buffer layer on a SiC substrate was systematically investigated. The ultra-thin 380 nm AlGaN/GaN/AlN had better crystalline quality, as characterized by AFM, XRD, and Hall measurements. Carrier distribution at high temperatures extracted from the *C*-*V* curves of Schottky diodes indicated that the non-buffer epi-structure could be favorable for improving 2DEG confinement. MIS-HEMTs on the non-buffer epi-structure almost exhibited the same performance in DC characteristics as the conventional epi-structure on the SiC substrates, which were much better than the current commercial structure on the Si substrate. The pulsed-*I*-*V* measurements demonstrated the advantage of the non-buffer design, which showed the lowest current collapse compared to the conventional structures on both the SiC and Si substrates. This was attributed to the improved crystalline quality, enhanced electron confinement, and significantly reduced deep-level traps induced by the thick buffer doping. These characteristics made the non-buffer epi-structure on the SiC substrate an excellent candidate in AlGaN/GaN power HEMTs applications.

## Figures and Tables

**Table 1:** Hall test results of the three epitaxial structures.

Epi-Structures	*R*_sheet_ (Ω/sq)	*μ*_2DEG_ (cm^2^/V·s)	*n*_s_ (10^12^ cm^−2^)
Non-buffer epi-structure on SiC	292	2287	9.4
Conventional epi-structure on SiC	284	2245	9.8
Conventional epi-structure on Si	365	1835	9.3

## Data Availability

Not applicable.
